# Effect of tooth discolouration severity on the efficacy and colour stability of two different trayless at-home bleaching systems

**DOI:** 10.15171/joddd.2018.019

**Published:** 2018-06-20

**Authors:** R. Banu Ermis, Esra Uzer CELIK, Gul YILDIZ, Basak YAZKAN

**Affiliations:** ^1^Department of Restorative Dentistry, Faculty of Dentistry, Suleyman Demirel University, Isparta, Turkey Turkey; ^2^Department of Restorative Dentistry, Faculty of Dentistry, Izmir Katip Celebi University, Izmir, Turkey; ^3^Department of Restorative Dentistry, Faculty of Dentistry, Recep Tayyip Erdogan University, Rize, Turkey; ^4^Department of Restorative Dentistry, Faculty of Dentistry, Pamukkale University, Denizli, Turkey

**Keywords:** Color stability, tooth bleaching, tooth discolouration severity, trayless bleaching system

## Abstract

***Background.*** The use of trayless at-home bleaching agents in darker teeth is raising some concerns due to their unknown efficacy. The purpose of this in vitro study was to evaluate the effect of tooth discolouration severity on the efficacy and colour stability of two different trayless at-home bleaching systems.

***Methods.*** Ninety enamel-dentin samples were divided into two groups: lighter tand darker teeth. The teeth in each group were further divided into three groups (n=15): (1) tray-based system with 10% carbamide peroxide (Opalescence Oh) (control group); (2) a tray applied whitening membrane with 10% hydrogen peroxide (Treswhite Supreme); and (3) a whitening pen with 22% carbamide peroxide (Hollywood Smiles). After bleaching, the teeth were stained for 9 days using red wine. Colour readings before bleaching treatment were determined using a dental spectrophotometer and were repeated 24 h after bleaching treatment and 24 h after staining process. Analysis of variance, Bonferroni and Dunnett C tests were used at 5% significance level.

***Results.*** No differences were observed between the bleaching efficacy of Treswhite Supreme and Opalescence Oh in the lighter teeth, while Opalescence Oh had the best bleaching efficacy in the darker teeth (p<0.05). Hollywood Smiles had the worst bleaching efficay but the best color stability in both lighter and darker teeth (p<0.05). No differences were observed between the color stability values of Treswhite Supreme and Opalescence Oh.

***Conclusion.*** This study suggested that both the tooth discolouration severity and the type of trayless system used affected the bleaching efficacy, whereas only the type of trayless system affected the color stability.

## Introduction

Tooth bleaching is an effective and non-invasive procedure for improving the color of teeth.^1,2^ Today, the clinician has different choices, including a variety of at-home tray-based bleaching agents with low concentration of hydrogen peroxide or carbamide peroxide.^2^ In-office bleaching systems, on the other hand, use a high concentration of hydrogen peroxide or carbamide peroxide, applied by a clinician in a dental office.^1^ Previous studies have shown both methods to be satisfactory and safe.^3,4^

The latest trend in bleaching procedures is to get whiter teeth at home in a shorter time, and the trayless bleaching systems are the predominant modalities that help achieve this end.^5-8^ Trayless bleaching systems are less expensive and easier to use than the traditional professional bleaching systems.^5-8^ The various forms of trayless products include gels, rinses, dentifrices, strips and paint-on films or pens with different levels of hydrogen peroxide or carbamide peroxide.^7,8^

Although patients may use these trayless systems without consulting a professional, the absence of professional supervision increases the risk of misuse or overuse of the products.^1,5^ Therefore, adverse effects such as tooth sensitivity, tissue irritation, and structural changes in the enamel may be more commonly encountered with such systems.^1,5^ There is also a large deviation in the efficacy of such products that depends on the concentration, type of bleaching agent and application procedure.^9-12^ Lack of information regarding their efficacy, long-term color stability and potential adverse effects has raised some concerns over the routine use of these products.

One of the most important factors that might affect the efficacy and color stability of bleaching treatments is the severity of tooth discoloration. Since longer application times led to greater improvements in shade,^11,12^ trayless bleaching products with short application times compared to professional bleaching systems might not be the right choice for bleaching darkly stained teeth. However, there is a lack of evidence regarding the efficacy and color stability of bleaching systems with respect to the severity of tooth discoloration.

The aim of this study was to compare the bleaching efficacy and color stability of two different trayless at-home bleaching systems: a tray-applied whitening membrane and a whitening pen with a tray-based at-home bleaching system and to determine whether these parameters are affected by the severity of tooth discoloration. The null hypotheses tested were: (1) There is no difference in the bleaching efficacy of different at-home bleaching systems on lightly and darkly stained teeth; and (2) Severity of tooth discoloration and the type of trayless at-home bleaching systems used have no effect on the color stability of the treated teeth.

## Methods

### Study design and tooth samples

**Figure 1:**
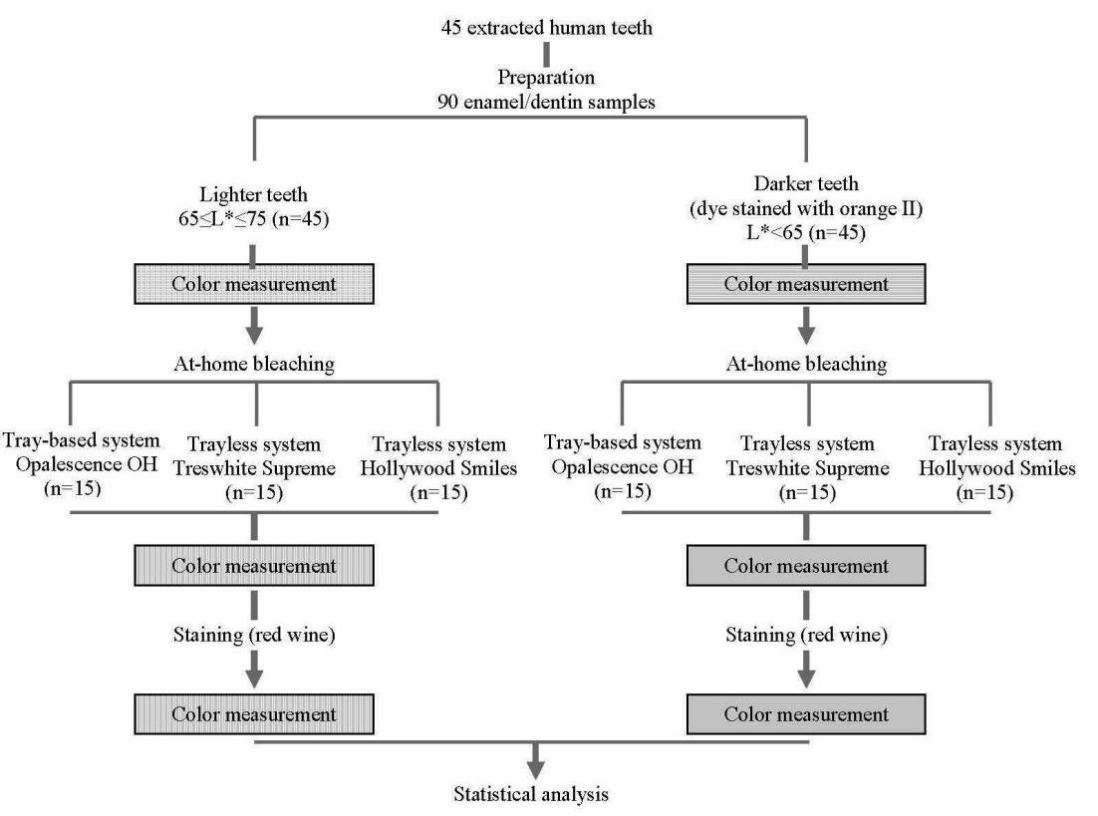
Group assignment and overview of the study method.

Caries-free extracted human molars, which were collected under the approval of Institutional Ethics Committee, were divided into two groups: lighter (65≤L*≤75) or darker (L*<65) teeth. A dye solution was used to stain the teeth in the darker group. Thereafter, the teeth in both groups were further divided into three groups for treatment with different at-home bleaching products. After bleaching, the teeth in all the groups were subjected to staining. A quantitative method was used to measure the color changes. An overview of the study method is illustrated in Figure 1.

In this study, 45 caries-free human molars were used. All the teeth were hand-scaled to remove any residual tissue, cleaned with a rubber cup and a fluoride-free pumice powder, stored in 2% sodium azide (Merck KGaA, Darmstadt, Germany) in distilled water at 4°C, and used within 1 month of extraction. The teeth were bisected longitudinally in a mesiodistal direction using a slow-speed diamond saw (Microcut125, Metkon, Bursa, Turkey) under continuous water cooling to obtain 90 enamel‒dentin samples. The color values of the tooth samples were measured using a dental spectrophotometer (SpectroShade, MHT Optic Research AG, Niederhasli, Switzerland). Initially, all the tooth samples had L* values between 65 and 75. Half of these samples were randomly selected to be stained with the Orange II dye solution (Sigma-Aldrich Co., St. Louis, MO, USA) for obtaining darker teeth.^13^ The enamel surfaces of these samples were first cleaned with a pumice paste using prophylaxis polishing cups (TPC Advanced Technology, CA, USA). The dentin surfaces were etched for 60 s using 32% orthophosphoric acid (Uni-etch, Bisco, Inc., Schaumburg IL, USA) and rinsed with water for 30 s. The Orange II dye was diluted with distilled water to a concentration of 0.3 mM. The samples were immersed in the dye solution and measured periodically using the dental spectrophotometer until the required degree of staining was achieved,^13^ as determined by L*<65.^14^ Prior to each measurement, the enamel surfaces were polished using a pumice paste and polishing cups to remove extrinsic stains.

### Groups

The lighter (65≤L*≤ 75) and darker (L*<65) tooth groups of 45 samples each were further divided into 3 groups (n=15) randomly for treatment with different at-home bleaching products as follows: (1) a tray-based system using 10% carbamide peroxide (Opalescence Oh, Ultradent, South Jordan, UT, USA) (control group); (2) a trayless system in the form of a tray-applied whitening membrane using 10% hydrogen peroxide (Treswhite Supreme, Ultradent); and (3) a trayless system in the form of a whitening pen using 22% carbamide peroxide (Hollywood Smiles, Hollywood Smiles UK Ltd, Glasgow, UK) (Table 1).

**Table 1:** At-home bleaching products

**Material/Manufacturer**	**Type/Concentration**	**Application Protocol**
Opalescence Oh (Ultradent, South Jordan, UT, USA)	Tray-based system (control) 10% carbamide peroxide	8 hours once daily, 14 days
Treswhite Supreme (Ultradent, South Jordan, UT, USA)	Trayless system 10% hydrogen peroxide	30 minutes twice daily, 14 days
Hollywood Smiles (Hollywood Smiles, Glasgow, UK)	Trayless system 22% carbamide peroxide	10 minutes once daily, 14 days

The dentin and root surfaces of all the samples were covered with two layers of colorless nail varnish. Before commencing the bleaching treatment, the L*, a*, and b* values of each sample were determined using a dental spectrophotometer.

### Tray-based system with 10% carbamide peroxide gel

The enamel surfaces of the samples were air-dried; then 10% carbamide peroxide gel (Opalescence Oh) was applied and left on for 8 h/day for 14 days according to the manufacturer’s instructions.

### Trayless system with 10% hydrogen peroxide gel

To use the whitening membrane (Treswhite Supreme) for a single tooth sample, the tray was cut into pieces according to the size of the enamel surface of each sample. The enamel surfaces were then air-dried, and the thin membrane was applied to each sample twice daily and left on for 30 min for 14 days according to the manufacturer’s instructions.

### Trayless system with 22% carbamide peroxide gel

The enamel surfaces of the samples were air-dried; then the whitening pen (Hollywood Smiles) was applied to the samples and left on for 10 min/day for 14 days according to the manufacturer’s instructions.

All the samples were placed in a tray with moist cotton in an incubator at 37°C and 100% relative humidity during bleaching. During the resting periods, they were stored in artificial saliva (0.33 g of KH_2_PO_4_, 0.34 g of Na_2_HPO_4_, 1.27 g of KCl, 0.16 g of NaSCN, 0.58 g of NaCl, 0.17 g of CaCl_2_, 0.16 g of NH_4_Cl, 0.03 g of glucose, 0.2 g of urea, 0.002 g of ascorbic acid and 2.7 g of mucin in 1000 mL of distilled water) in an incubator at 37°C. After the bleaching process was completed, all the samples were polished using aluminum oxide polishing discs (fine to super fine; Sof-Lex, 3M ESPE, St. Paul, MN, USA). A sodium fluoride gel (2%; Sultan Topex Neutral Fluoride gel, Englewood, NJ, USA) was applied to the samples for 5 min. The color readings were repeated 24 h after completion of bleaching.

### Staining

The staining process was initiated after obtaining the color readings of the bleached samples. The samples were stained for 10 min/day using red wine (Şirazettin, Cumartesi, Turkey) for 9 days.^15,16^ At all other intervals, the samples were stored in artificial saliva in an incubator at 37°C. After the staining process was completed, extrinsic stains were removed with a pumice paste using polishing cups. The color readings were repeated after 24 h.

### Data analysis

The color change for intervals between the baseline and after bleaching (DE1) and between the interval after bleaching and after staining (DE2) was calculated using the following formula: DE=[(DL*)^2^+(Da*)^2^+(Db*)^2^]^1/2^, where DE=color change; DL=L_final_-L_initial_; Da=a_final_-a_initial_ and Db=b_final_-b_initial_.

### Statistical analysis

### Results

**Figure 2:**
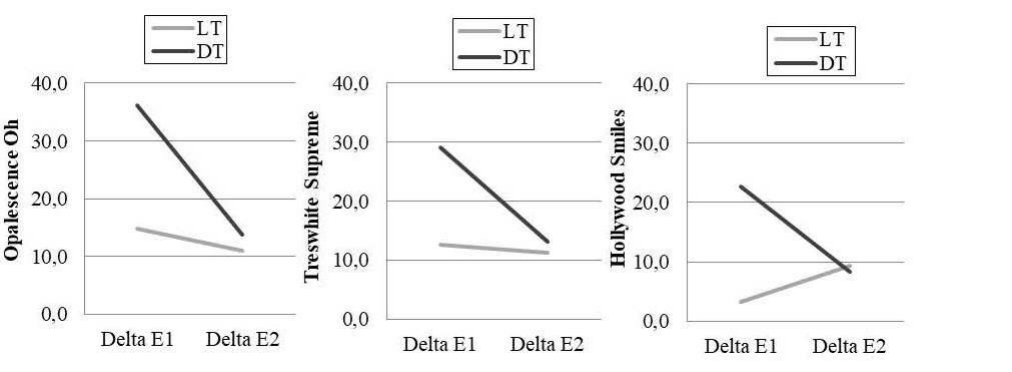
Graphic representation of ∆E1 and ∆E2 values of different bleaching systems for lighter (LT) and darker teeth (DT).

Variations in colorimetric measurements (L*, a*, and b*) for lighter and darker teeth, before and after bleaching, as well as after staining, and their statistical comparisons are presented in Tables 2a and 2b. DE values for the tested bleaching systems, in lighter and darker teeth, and their statistical comparisons are presented in Table 3. Statistically significant differences are shown in Tables 4a and 4b. Figure 2 presents the comparison of DE values between lighter and darker teeth.

**Table 2a:** Variations in colorimetric measurements (L*a*b*±SD) of lighter teeth at three time intervals. Means with same superscripts in a column within the same group are not significantly different at P<0.05

L*	Opalescence Oh	Treswhite Supreme	Hollywood Smiles
**Before Bleaching**	71.3^A^±3.6	71.1^a^±4.0	69.2^X^±2.6
**After Bleaching**	82.3^B^±1.9	80.5^b^±2.3	70.9^X^±2.3
**After Staining**	71.4^A^±2.0	69.5^a^±2.3	62.8^Y^±2.7
a*	Opalescence Oh	Treswhite Supreme	Hollywood Smiles
**Before Bleaching**	4.0^A^±1.8	3.8^a^±1.9	5.4^X^±1.5
**After Bleaching**	1.1^B^±0.6	1.2^b^±1.1	4.1^X^±1.3
**After Staining**	2.1^B^±0.7	2.7^a^±1.0	8.8^Y^±1.7
b*	Opalescence Oh	Treswhite Supreme	Hollywood Smiles
**Before Bleaching**	22.3^A^±3.4	21.5^a^±3.9	22.7^X^±2.4
**After Bleaching**	13.7^B^±2.9	14.0^b^±4.0	21.2^X^±2.5
**After Staining**	14.8^B^±2.8	14.9^b^±3.7	21.3^X^±2.3

**Table 2b:** Variations in colorimetric measurements (L*a*b*±SD) of darker teeth at three time intervals. Means with same superscripts in a column within the same group are not significantly different at P<0.05

L*	Opalescence Oh	Treswhite Supreme	Hollywood Smiles
**Before Bleaching**	61.5^A^±3.2	62.9^a^±2.8	60.7^X^±3.4
**After Bleaching**	79.5^B^±3.3	77.2^b^±2.7	70.2^Y^±3.3
**After Staining**	67.3^C^±3.2	65.5^a^±5.1	63.0^X^±2.5
a*	Opalescence Oh	Treswhite Supreme	Hollywood Smiles
**Before Bleaching**	24.4^A^±3.7	21.8^a^±4.2	22.6^X^±2.8
**After Bleaching**	8.4^B^±3.7	7.6^b^±2.6	6.7^Y^±2.4
**After Staining**	13.6^C^±2.3	11.5^c^±3.1	10.1^Z^±2.6
b*	Opalescence Oh	Treswhite Supreme	Hollywood Smiles
**Before Bleaching**	40.1^A^±1.8	34.4^a^±6.5	33.6^X^±4.2
**After Bleaching**	13.4^B^±4.7	13.7^b^±4.5	21.3^Y^±4.5
**After Staining**	13.8^B^±2.8	12.3^b^±2.3	21.6^Y^±3.6

The changes in L*, a* and b* values throughout bleaching and staining were analyzed using a linear mixed model. The type of bleaching system, tooth color and measurement time were considered as fixed factors. The interactions between these factors were assessed using nested ANOVA (measurement time [tooth discoloration severity {bleaching system}]) and the Bonferroni test was used for post hoc comparisons (Tables 2a and 2b).

A general linear model was used to evaluate the effects of the type of bleaching system and the tooth discoloration severity on DE1 and DE2. The interaction between the type of bleaching system and tooth discoloration severity was analyzed using the two-way ANOVA. Bonferroni test was used when equal variation was assumed, and the Dunnett C test was used when equal variance was not assumed (Table 3). For each bleaching system, DE1 and DE2 values were compared between the lighter and darker samples using independent t-test (Figure 2). For all the tests, the probability level for statistical significance was set at a=0.05.

**Table 3:** ∆E values (±SD) of lighter and darker teeth for each tested bleaching system. Means with same superscripts in a row within the same group are not significantly different at P<0.05

**Before Bleaching/After Bleaching**	Opalescence Oh	Treswhite Supreme	Hollywood Smiles
**Lighter teeth (∆E1-LT)**	14.8^A^±2.9	12.6^A^±2.9	3.3^B^±3.1
**Darker teeth (∆E1-DT)**	36.1^a^±8.1	29.1^b^±7.5	22.7^c^±4.0
**After bleaching/After staining**	Opalescence Oh	Treswhite Supreme	Hollywood Smiles
**Lighter teeth (∆E2-LT)**	11.1^X^±2.0	11.2^X^±2.0	9.4^Y^±2.1
**Darker teeth (∆E2-DT)**	13.8^a^±2.5	13.2^a^±4.8	8.3^b^±2.8

After bleaching, a significant increase in L* values and a significant decrease in a* and b* values were observed for Opalescence Oh and Treswhite Supreme in both lighter and darker teeth. Hollywood Smiles exhibited significant differences in L*, a*, and b* values in darker teeth only. The staining of samples produced a clear reduction in L* values and an increase in a* values for both lighter and darker teeth. After staining, no significant difference was observed in b* values for all the bleaching systems. For lighter teeth treated with Opalescence Oh, there was also no significant difference in a* values after staining.

**Table 4a:** aP-values from ANOVA and Bonferroni test; P-values <0.05 indicate significant difference

		Opalescence Oh			Treswhite Supreme			Hollywood Smiles		
		L*	a*	b*	L*	a*	b*	L*	a*	b*
**Lighter**	**Before bleaching/After bleaching**	0.000	0.000	0.000	0.000	0.000	0.000	0.196	0.071	0.254
	**Before bleaching/After staining**	1.000	0.000	0.000	0.081	0.095	0.000	0.000	0.000	0.350
	**After bleaching/After staining**	0.000	0.059	0.395	0.000	0.015	1.000	0.000	0.000	1.000
**Darker**	**Before bleaching/After bleaching**	0.000	0.000	0.000	0.000	0.000	0.000	0.000	0.000	0.000
	**Before bleaching/After staining**	0.000	0.000	0.000	0.165	0.000	0.000	0.160	0.000	0.000
	**After bleaching/After staining**	0.000	0.000	1.000	0.000	0.009	1.000	0.000	0.002	1.000

**Table 4b:** P-values of comparisons between DE values of materials for lighter and darker teeth are presented in the lower left (below dashes) and upper right half (above dashes) of the table, respectively. P-values <0.05 indicate significant difference

**Before bleaching/After bleaching**	Opalescence Oh	Treswhite Supreme	Hollywood Smiles
**Opalescence Oh**	-	0.021	0.000
**Treswhite Supreme**	0.162	-	0.039
**Hollywood Smiles**	0.000	0.000	-
**After bleaching/After staining**	Opalescence Oh	Treswhite Supreme	Hollywood Smiles
**Opalescence Oh**	-	1.000	0.000
**Treswhite Supreme**	1.000	-	0.001
**Hollywood Smiles**	0.049	0.044	-

After bleaching, while no significant difference was observed between DE1 values for lighter teeth treated with Opalescence Oh (DE1-LT: 14.8) and Treswhite Supreme (DE1-LT: 12.6), Opalescence Oh (DE1-DT: 36.1) was significantly better than Treswhite Supreme (DE1-DT: 29.1) in darker teeth. For Hollywood Smiles, DE1 values were significantly lower than those of Opalescence Oh and Treswhite Supreme after bleaching in both lighter (DE1-LT: 3.3) and darker teeth (DE1-DT: 22.7). After staining with red wine, lighter and darker tooth samples bleached with Opalescence Oh (DE2-LT: 11.1, DE2-DT: 13.8) and Treswhite Supreme (DE2-LT: 11.2, DE2-DT: 13.2) demonstrated similar color stability and were more susceptible to stains than tooth samples bleached with Hollywood Smiles (DE2-LT: 9.4, DE2-DT: 8.3).

When each bleaching system was analyzed in regard to DE values between lighter and darker teeth following bleaching (DE1-LT vs. DE1-DT) and staining (DE2-LT vs. DE2-DT), all the products exhibited DE1-DT values significantly higher than DE1-LT values (P=0.000). Following staining, no significant difference was reported between DE2-LT and DE2-DT for Treswhite Supreme (P=0.175) and Hollywood Smiles (P=0.220). However, DE2-DT values were higher than DE1-LT values for Opalescence Oh (P=0.041).

## Discussion

### Comparisons between bleaching systems

Treswhite Supreme and Opalescence Oh yielded similar bleaching efficacy for lighter teeth. However, the efficacy of Treswhite Supreme was inferior to Opalescence Oh for bleaching of darker teeth, despite having higher concentration of hydrogen peroxide. This difference might be explained by the lower number of oxidizing ions that can migrate through hard tissues during the recommended application time for Treswhite Supreme. The application time of a bleaching agent is determined based on the concentration and kinetic release of hydrogen peroxide during the application of the product; 100% hydrogen peroxide release is expected within the manufacturers’ recommended application time.^17^ However, according to a study by da Silva Marques et al^16^ Treswhite Supreme failed to release the entirety of its hydrogen peroxide content in 60 min. Therefore, a longer treatment time might be needed for Treswhite Supreme to show efficacy similar to Opalescence Oh in darker teeth. In addition, variations in matrix modification and the extent of gel impregnation might also account for the difference in their bleaching performance. In an in vitro study, Dietschi et al^18^ observed no difference between the efficacy of Treswhite and Opalescence for bleaching lighter teeth (L*>65). This finding is consistent with the present study.

Hollywood Smiles showed the lowest bleaching efficacy in both lighter and darker teeth. Even though this system revealed a statistically significant amount of increase in L* values and decrease in a* and b* values in darker teeth after bleaching, no significant changes in L*, a* and b* values were recorded in lighter samples after the same process. Gambarini et al^10^ reported an improvement of 4.5 shades using 5.9% hydrogen peroxide. A study that used 6% hydrogen peroxide reported an improvement of only 1.02 shades.^11^ One product with 18% carbamide peroxide improved the tooth color by 3.8‒5.5 shades in different studies, depending on the treatment time.^12^ Differences in the active agent type, concentration and application time might account for the variations of different paint-on products seen in these study results. On the other hand, paint-on whiteners demonstrated less improvement in color compared to whitening strips, tray-applied whitening membranes and tray-based systems.^18,19^ The lower bleaching efficacy by these products might be attributed to the shorter contact time with the teeth. Notably, carbamide peroxide requires more time to release its entire hydrogen peroxide content. Even paint-on whiteners containing 6% hydrogen peroxide have a recommended treatment time of 10 min or longer, so the manufacturer’s recommended 10-min treatment time for Hollywood Smiles might not be long enough for a carbamide peroxide-containing product.

In this study, the efficacy of the bleaching systems depended on the severity of discoloration, because the DE1 values of all the bleaching agents were higher for the darker teeth than their lighter counterparts. As the darker teeth had much lower L* and much higher a* and b* values before bleaching, the color change after bleaching was greater; therefore, it was easier to detect. A previous research also reported the best bleaching results for brown- and yellow-stained teeth.^20^ Therefore, in laboratory and clinical studies, teeth of similar shades should be used to evaluate the differences between bleaching agents; otherwise, products tested on teeth of darker shades might produce significantly greater color differences, owing to the lower L* and higher a* and b* values prior to bleaching.

### Color stability

Tooth bleaching therapy might negatively affect the tooth structure due to the oxidative action, pH or the composition of the bleaching agent in varying degrees, depending on the type of bleaching system.^21^ Thus, the long-term performance of bleaching treatments is debatable, and in many cases, some degree of rebound effect has been observed within days or weeks following the bleaching procedure.^22^ Oxidative action by hydrogen peroxide can cause structural and permeability changes and surface porosity in the enamel surface.^23,24^ Furthermore, coloring pigments might accumulate on the rough surface, and a rough enamel surface with pores or superficial defects might discolor easily.^25^

In this study, red wine was preferred for staining the teeth after bleaching. A variety of substances, including tea, coffee, chlorhexidine and red wine have been used to stain the bleached teeth when the susceptibility of bleached teeth to staining has been evaluated.^25-28^ Although all the materials demonstrated a staining effect in these previous studies, bleached teeth were shown to be more susceptible to red wine stains.^26^

Hollywood Smiles exhibited the best color stability (the lowest DE2 values), while the color stability of Treswhite Supreme and Opalescence Oh were comparable for both lighter and darker teeth. These results might be attributed to the low hydrogen peroxide concentration and shorter treatment time of Hollywood Smiles. It has been reported in a number of articles that bleaching of enamel increases its susceptibility to extrinsic stains.^26,28,29^ In a previous in vitro study, 35% hydrogen peroxide was found to cause greater tendency for staining compared to 16% carbamide peroxide.^30^ Scanning electron microscopic evaluations have also revealed that surface morphological alterations increase with higher concentrations of hydrogen peroxide and longer treatment times.^31,32^

In this study, the severity of discoloration did not affect the color stability of the teeth treated with the different bleaching systems. These results support the idea that staining after bleaching is promoted by structural changes in the enamel caused by the bleaching agents, and is not due to the perceived lower color stability of darker teeth.

## Conclusion

The tray-applied whitening membrane might be an effective alternative to the tray-based at-home bleaching system for use on lighter teeth. However, the tray-based at-home bleaching system might be the best choice for treating darker teeth. The whitening pen demonstrated the lowest bleaching efficacy in both lighter and darker teeth. The bleaching efficacy was better in darker teeth than in the lighter ones in all the bleaching systems tested. Thus, the first hypothesis that bleaching effect is not dependent on the tooth discoloration severity and the type of at-home bleaching system used was rejected.

In relation to the color stability, tray-applied whitening membrane and tray-based at-home bleaching system exhibited similar performance. The color stability was highest in teeth treated with the whitening pen among the products tested. These results were not affected by tooth discoloration severity. Thus, the second hypothesis that severity of tooth discoloration and the type of trayless at-home bleaching systems used have no effect on the color stability of teeth treated was partially accepted.

## Acknowledgments

None.

## Authors’ contributions

RBE and EUC contributed to the conception, design, data analysis and interpretation, and drafted the manuscript. GY and BY contributed to data collection, contributed to data collection. All authors contributed to the critical revision of the paper and have read and approved the final manuscript.

## Funding

This study was supported by the Suleyman Demirel University Scientific Research Projects Foundation (2499-M-10).

## Competing interests

The authors declare no competing interests with regards to the authorship and/or publication of this article.

## Ethics approval

This study was conducted with the approval of the Ethical Committee of İzmir Kâtip Çelebi University, İzmir.
